# The Role of Alpha-Synuclein Oligomerization and Aggregation in Cellular and Animal Models of Parkinson’s Disease

**DOI:** 10.1371/journal.pone.0038545

**Published:** 2012-06-12

**Authors:** Oi Wan Wan, Kenny K. K. Chung

**Affiliations:** Division of Life Science, State Key Laboratory of Molecular Neuroscience, The Hong Kong University of Science and Technology, Hong Kong, China; Hertie Institute for Clinical Brain Research and German Center for Neurodegenerative Diseases, Germany

## Abstract

α-synuclein (α-syn) is a synaptic protein in which four mutations (A53T, A30P, E46K and gene triplication) have been found to cause an autosomal dominant form of Parkinson’s disease (PD). It is also the major component of intraneuronal protein aggregates, designated as Lewy bodies (LBs), a prominent pathological hallmark of PD. How α-syn contributes to LB formation and PD is still not well-understood. It has been proposed that aggregation of α-syn contributes to the formation of LBs, which then leads to neurodegeneration in PD. However, studies have also suggested that aggregates formation is a protective mechanism against more toxic α-syn oligomers. In this study, we have generated α-syn mutants that have increased propensity to form aggregates by attaching a CL1 peptide to the C-terminal of α-syn. Data from our cellular study suggest an inverse correlation between cell viability and the amount of α-syn aggregates formed in the cells. In addition, our animal model of PD indicates that attachment of CL1 to α-syn enhanced its toxicity to dopaminergic neurons in an age-dependent manner and induced the formation of Lewy body-like α-syn aggregates in the substantia nigra. These results provide new insights into how α-syn-induced toxicity is related to its aggregation.

## Introduction

Parkinson’s disease (PD) is a common neurodegenerative disorder that is marked by the degeneration of dopaminergic neurons in the substantia nigra pars compacta (SNc) and the presence of Lewy bodies (LBs) [1]–[5]. LBs are cytoplasmic eosinophilic protein aggregates with α-synuclein (α-syn) as one of the major components [6], [7]. α-syn is a synaptic protein in which four mutations (A53T, A30P, E46K and gene triplication) have been found to cause an autosomal dominant form of PD [8]–[13]. The familial PD (FPD) linked point mutations and gene triplications in α-syn suggest that abnormal structure or excessive accumulation of α-syn can enhance its toxicity and lead to the degeneration of dopaminergic neurons in PD. In different studies, both wild type (WT) and mutant forms of α-syn have been shown to have a high propensity for forming oligomers and fibrils when incubated *in vitro,* while in α-syn transgenic animal model, the presence of α-syn aggregation is associated with neuronal degeneration. [4], [14]–[21]. These results suggest that the process of oligomerization, fibrillization and aggregation of α-syn are the culprits behind the neurodegeneration seen in PD [13], [19], [21]–[24]. However, some studies have also suggested that α-syn aggregates might be protective and oligomers and pre-fibrilliar α-syn are the toxic species responsible for neurodegeneration [25]. For instance, small molecule that facilitates α-syn inclusion formation or histone deacetylase inhibition that enhances enlarged α-syn inclusion formation provides protection in cell against α-syn induced toxicity [26]–[28]. A recent study has also shown that α-syn mutants that have reduced propensity to form fibrils and aggregates have increased toxicity [29]. In this study, we used another approach to determine if α-syn aggregation is directly related to its cellular toxicity by generating α-syn mutants that have a higher propensity to form intracellular aggregates. We used a 16 amino acids peptide called CL1, which has been shown to destabilize GFP for proteasomal degradation and enhance the GFP aggregation [30], [31]. We generated α-syn mutants by attaching the CL1 peptide to the C-terminal and studied how the enhancement of α-syn aggregation affected its toxicity in cellular and animal models. Our results provide new insights into how α-syn-induced toxicity is related to its aggregation.

## Results

### CL1 Sequesters α-syn to the Detergent-insoluble Fraction of SHSY5Y Cells

**Figure 1:**
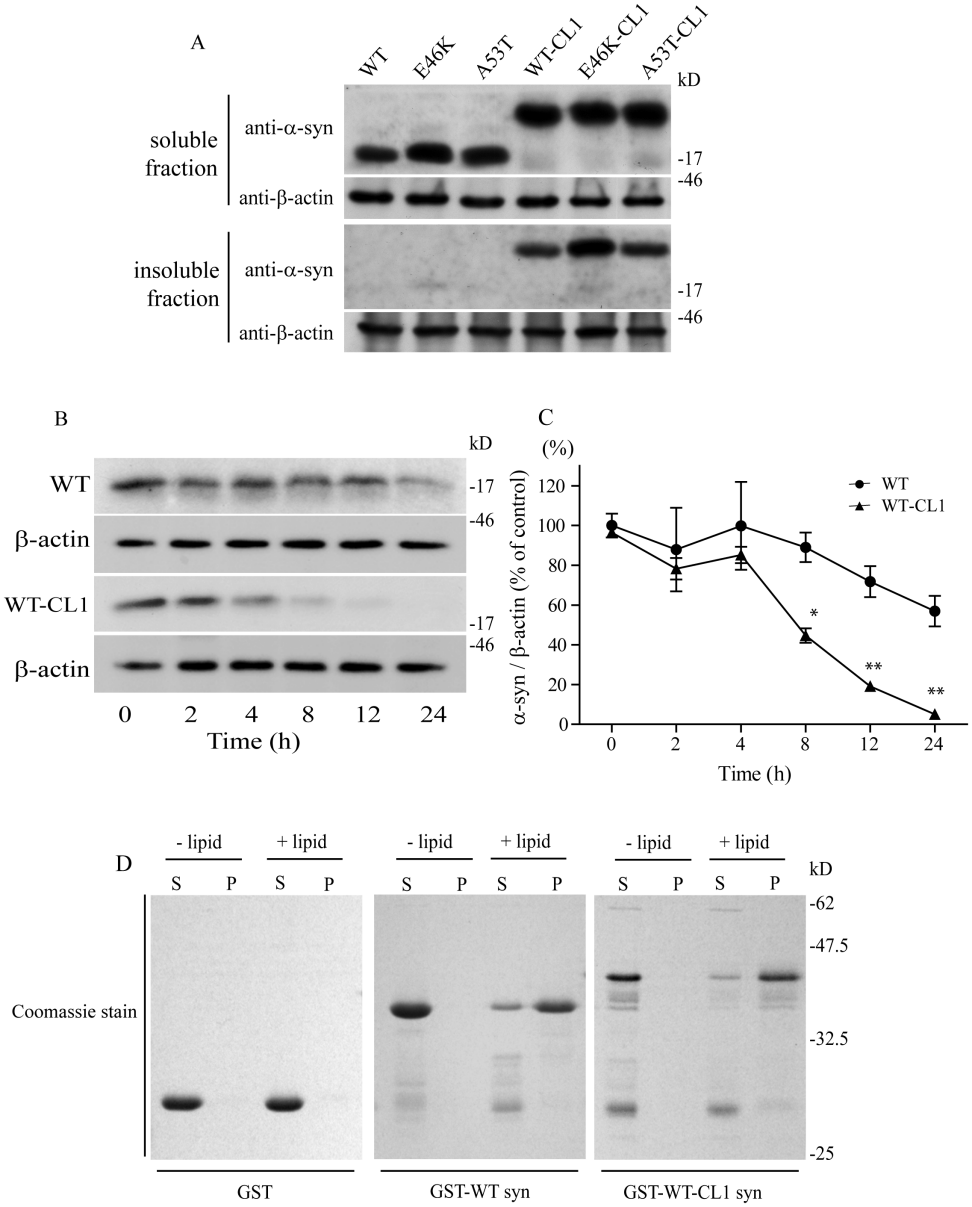
CL1 sequesters α-syn to the detergent-insoluble fraction of SHSY5Y cells. (A) SHSY5Y cells expressing WT, E46K, A53T, WT-CL1, E46K-CL1 or A53T-CL1 α-syn were fractionated to soluble and insoluble fractions and analyzed by Western blot. Attachment of CL1 to WT or mutant α-syn reduced their solubility in the SHSY5Y cells. (B) SHSY5Y cells expressing WT or WT-CL1 α-syn were subjected to cycloheximide pulse chase analysis. Cells were harvested at different time points and subjected to Western blot analysis. The degradation of WT-CL1 α-syn was enhanced when compared to WT α-syn. (C) Three independent cycloheximide pulse chase experiments were performed and the levels of WT or WT-CL1 α-syn were quantified using optical densitometry (*p<0.05; **p<0.01). (D) GST, GST-WT α-syn or GST-WT-CL1 α-syn was mixed with or without liposome and subjected to lipid sedimentation assay. The samples were then analyzed by SDS-PAGE with Coomassie stain. Attachment of CL1 to α-syn did not affect its lipid binding affinity.

To establish a model that could promote the oligomerization and aggregation of α-syn, we generated wild type (WT) and mutant α-syn constructs fused with a 16 amino acid peptide CL1 at the C-terminal and characterized their solubility in the SHSY5Y cells. WT α-syn (WT), E46K α-syn (E46K), A53T α-syn (A53T), WT α-syn with CL1 (WT-CL1), E46K α-syn with CL1 (E46K-CL1) and A53T α-syn with CL1 (A53T-CL1) were expressed in SHSY5Y cells and their cellular localization was determined by extraction with soluble and insoluble fractionating buffers. Attachment of CL1 in WT and mutant α-syn enhanced their localization to the insoluble fractions of the cells (Figure 1A). To rule out the possibility that the accumulation of WT and mutant α-syn with CL1 in the insoluble fraction was caused by the extended half-life of α-syn in the cells, we performed a cycloheximide pulse chase experiment and monitored the degradation of WT and WT-CL1 α-syn. We found that both WT and WT-CL1 α-syn levels were significantly reduced within 24 h (Figure 1 B & C) (F = 14.39; p<0.0001, two way ANOVA). In addition, WT-CL1 α-syn was degraded significantly faster than WT (Figure 1B & C) (F = 25.93; p<0.0001, two way ANOVA). This result suggested that increased localization of WT and mutant α-syn with CL1 to the insoluble fraction was not caused by their extended half-life in the SHSY5Y cells. One of the characteristic of α-syn is its ability to bind to lipids. To determine if the attachment of CL1 to α-syn would affect its lipid binding affinity, we used a lipid sedimentation assay and found that both WT and WT-CL1 α-syn could bind to liposomes prepared from bovine brain (Figure 1D), suggesting that CL1 did not alter the lipid-binding affinity of α-syn.

### CL1 Enhances the Oligomerization and Aggregation of α-syn

**Figure 2:**
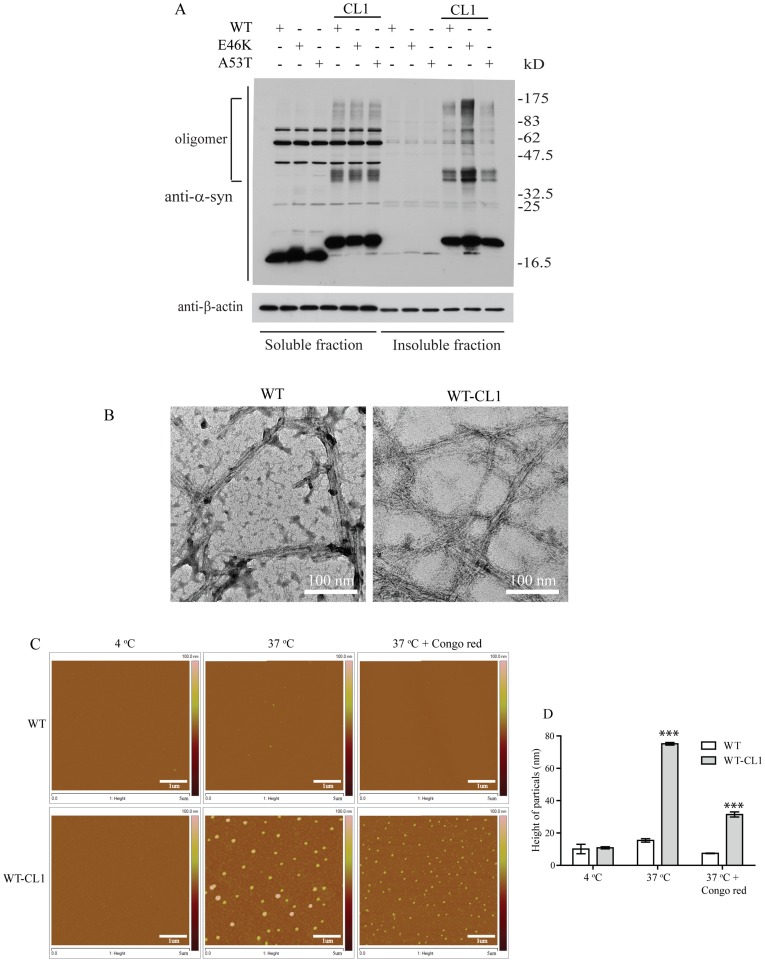
CL1 enhances the oligomerization and aggregation of α-syn. (A) SHSY5Y cells expressing WT, E46K, A53T, WT-CL1, E46K-CL1 or A53T-CL1 α-syn were fractionated to soluble and insoluble fractions and analyzed by Western blot. Attachment of CL1 to WT or mutant α-syn enhanced the formation of SDS-resistant α-syn oligomers. (B) Recombinant WT or WT-CL1 α-syn were incubated at 37°C for 2 weeks and then subjected to EM analysis. Both WT and WT-CL1 α-syn were able to form fibrils. (C) Recombinant WT or WT-CL1 α-syn was incubated at 4°C or 37°C with or without Congo red for 1 month and then subjected to AFM analysis. CL1 enhanced the formation of α-syn aggregates and coincubation of Congo red reduced the size of the aggregates. (D) Quantification of the size of α-syn aggregates in (C) (***p<0.0001).

**Figure 3:**
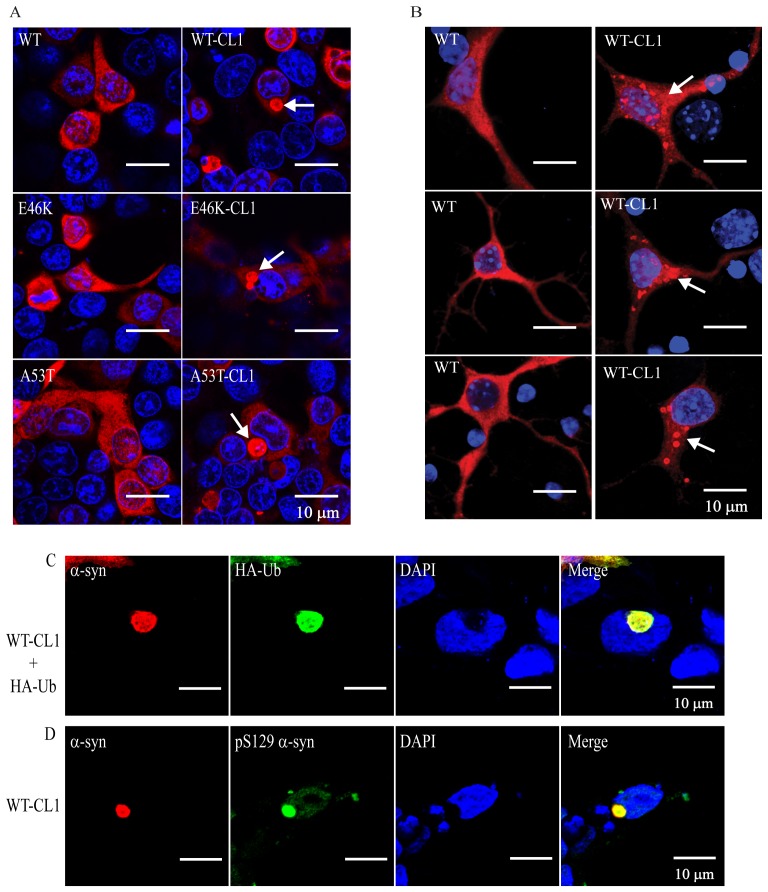
CL1 induces the formation of Lewy body-like aggregates in SHSY5Y cells and neurons expressing WT or mutant α-syn. (A) SHSY5Y cells expressing WT, E46K, A53T, WT-CL1, E46K-CL1 or A53T-CL1 α-syn were subjected to immunocytochemistry with anti-α-syn antibody. Lewy body-like perinuclear aggregates were observed in SHSY5Y cells expressing WT or mutant CL1 α-syn. (B) Primary neurons transfected with WT or WT-CL1 α-syn were subjected to immunocytochemistry with anti-human α-syn antibody. Prominent α-syn aggregates were observed in cortical neurons expressing WT-CL1 α-syn. (C) SHSY5Y cells expressing WT-CL1 and HA-Ub were subjected to immunocytochemistry with anti-α-syn and anti-HA antibodies. Perinuclear inclusion, which was positive for both α-syn and Ub was observed. (D) SHSY5Y cells expressing WT-CL1 were subjected to immunocytochemistry with anti-α-syn and anti-human α-syn phospho (Ser129) (anti-pS129α-syn). Perinuclear inclusion, which was positive for both α-syn and pS129α-syn was observed.

We found that CL1 sequesters α-syn to the detergent-insoluble fraction, therefore, we suspected that CL1 would also enhance α-syn oligomerization and aggregation. To test this, we expressed WT and mutant α-syn with and without CL1 in SHSY5Y cells to determine the presence of α-syn oligomers. As expected, enhanced formation of SDS-resistant oligomers were observed in both WT and mutant α-syn with CL1 (Figure 2A). Previous studies have shown that recombinant α-syn readily forms fibrils and aggregates after extended incubation at 37°C [22], [32], [33]. To determine if CL1 could affect fibrillization of α-syn, we incubated WT and WT-CL1 α-syn at 37°C for 2 weeks and analyzed the formation of fibrils by EM. We found that both WT and WT-CL1 α-syn were able to form fibrils under this condition (Figure 2B). To determine if CL1 could enhance α-syn aggregation, we incubated recombinant WT and WT-CL1 at 4°C and 37°C for 1 month and monitored the formation of protein aggregates by AFM. Because Congo red is known to inhibit α-syn aggregation, we also tested if CL1-enhanced α-syn aggregation could be inhibited by Congo red. CL1 significantly enhanced α-syn aggregation and this could be attenuated by Congo red (Figure 2 C & D). Consistent with some studies, the α-syn aggregates we observed under AFM were spherical rather than fibrillar [34], [35], as the morphology of α-syn aggregates examine under AFM is known to be affected by the physical conditions under which they are formed [36], [37]. To further determine how CL1 could affect α-syn aggregation in SHSY5Y cells, WT and mutant α-syn with or without CL1 expression in SHSY5Y cells were subjected to immunocytochemistry for α-syn. In SHSY5Y cells expressing WT, E46K and A53T α-syn, diffused cytoplasmic staining was observed (Figure 3A). However, in SHSY5Y cells expressing WT-CL1, E46K-CL1 and A53T-CL1 α-syn, large perinuclear α-syn inclusions that resemble LBs were found (Figure 3A). We also observed similar inclusions in cortical neurons expressing WT-CL1 α-syn, but not in neurons expressing WT α-syn (Figure 3B). In addition, we also found that some of these WT-CL1 inclusions in SHSY5Y cells were positive for ubiquitin (Ub) or phospho-Ser 129 of α-syn (Figure 3 C & D), supporting the notion that these α-syn inclusions highly resemble LBs. Overall, these results showed that CL1 could enhance the oligomerization and aggregation of α-syn.

### CL1 Enhances Aggregation and Cytotoxicity of α-syn

**Figure 4:**
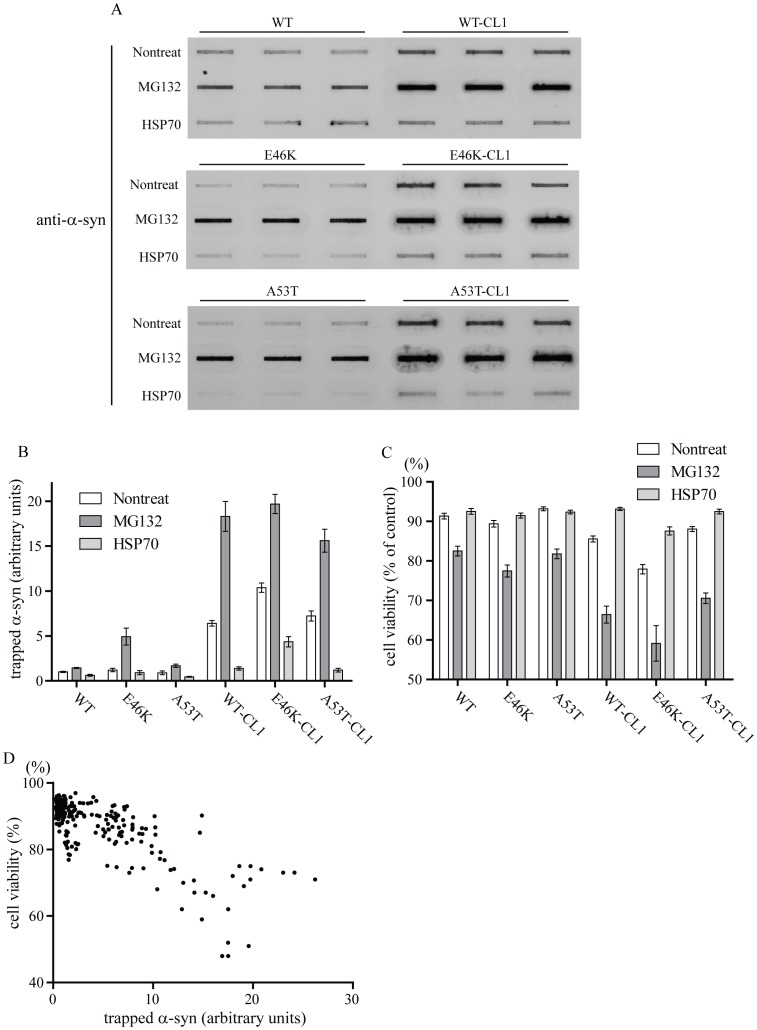
CL1 enhances aggregation and cytotoxicity of α-syn. (A) SHSY5Y cells expressing WT, E46K, A53T, WT-CL1, E46K-CL1 or A53T-CL1 α-syn under different conditions as indicated were harvested and total cell lysates were prepared and subjected to the filter retardation assay. (B) Quantification of trapped α-syn aggregates in filter retardation assay by optical densitometry in SHSY5Y cells expressing WT or mutant α-syn with or without CL1 in different conditions as indicated. (C) Cell viability was determined in the SHSY5Y cells expressing WT, E46K, A53T, WT-CL1, E46K-CL1 or A53T-CL1 α-syn under different conditions as indicated. (D) The cell viability was plotted against the amount of α-syn trapped for each individual data point for correlation analysis. There was a significant inverse correlation between cell viability and trapped α-syn (Pearson r = −0.89, p<0.0001). These results were based on three independent experiments.

Various studies have shown that α-syn aggregation is linked to an increased in cytotoxicity, however, the direct correlation between the level of α-syn aggregation and its induced cytotoxicity has not been well defined [22], [23]. We found that CL1 could enhance the aggregation of α-syn, therefore, we made use of this characteristic feature to determine if there was a direct correlation between α-syn aggregation and cell death. To quantify the level of α-syn aggregation, we used the filter retardation assay and measured the amount of trapped α-syn expressing in SHSY5Y cells. We used the trypan blue exclusion assay to determine the cell viability simultaneously. To facilitate our analysis, we included conditions that have been shown to enhance or reduce α-syn-induced cytotoxicity based on its aggregation. We included cells that were treated with proteasomal inhibitor (MG132), which enhanced α-syn aggregation, or cells that were coexpressed with HSP70, which inhibited α-syn aggregation. Attachment of CL1 significantly enhanced the aggregation of WT and mutant α-syn (Figure 4 A & B) (F = 578.8; p<0.0001, two way ANOVA) and reduced cell viability (Figure 4C) (F = 128.7; p<0.0001, two way ANOVA). Treatment of MG132 significantly enhanced the aggregation of WT and mutant α-syn (Figure 4 A & B) (F = 36.75; p<0.0001, two way ANOVA) and reduced cell viability (Figure 4C) (F = 186.1; p<0.0001, two way ANOVA). Similarly, treatment of MG132 significantly enhanced the aggregation of WT and mutant α-syn with CL1 (Figure 4 A & B) (F = 203.8; p<0.0001, two way ANOVA) and reduced cell viability (Figure 4C) (F = 160.9; p<0.0001, two way ANOVA). Coexpression of HSP70 significantly reduced the aggregation of WT and mutant α-syn (Figure 4 A & B) (F = 7.12; p<0.01, two way ANOVA), but did not change cell viability (Figure 4C) (F = 2.31; p = n.s.; two way ANOVA). Coexpression of HSP70 significantly reduced the aggregation of WT and mutant α-syn with CL1 (Figure 4 A & B) (F = 271.3; p<0.0001, two way ANOVA), and an increased in cell viability was observed (Figure 4C) (F = 118; p<0.0001, two way ANOVA). To determine if changes in α-syn induced cytotoxicity in association with coexpression of HSP70 or treatment of MG132 were not caused by changes in α-syn expression levels, we also examined the protein levels of WT and WT-CL1 α-syn. We did not observe any significant changes in the expression of WT or WT-CL1 α-syn under different conditions (Figure S1 A & B). These results showed that enhanced aggregation of α-syn through the attachment of CL1 or treatment with MG132 was associated with an increased in α-syn-induced cytotoxicity. In contrast, coexpression of HSP70 reduced aggregation of α-syn and enhanced cell viability. This relationship between the level of aggregation and α-syn-induced cytotoxicity was particularly prominent in SHSY5Y cells expressing WT or mutant α-syn with CL1. To further confirm that this correlation was significant, we performed a correlation analysis for individual data points and found that there was a significant inverse correlation between cell viability and the amount of trapped α-syn (Figure 4D) (Pearson r = −0.89, p<0.0001). To confirm that CL1 enhanced aggregation and increased toxicity of α-syn was specific to α-syn and not caused by CL1, we generated β-syn-CL1 and Htt-Q23-CL1 to determine if attachment of CL1 to β-syn and Htt-Q23, which are both non-cytotoxic to SHSY5Y cells and have similar molecular weight as α-syn, could enhance their aggregation and affect their cytotoxicity. Attachment of CL1 to β-syn and Htt-Q23 enhanced their aggregation (Figure S1C), but did not affect their cytotoxicity (Figure S1D), suggesting that enhanced aggregation and cytotoxicity was specific to α-syn.

### Increased α-syn Oligomerization is Linked to its Cytotoxicity

**Figure 5:**
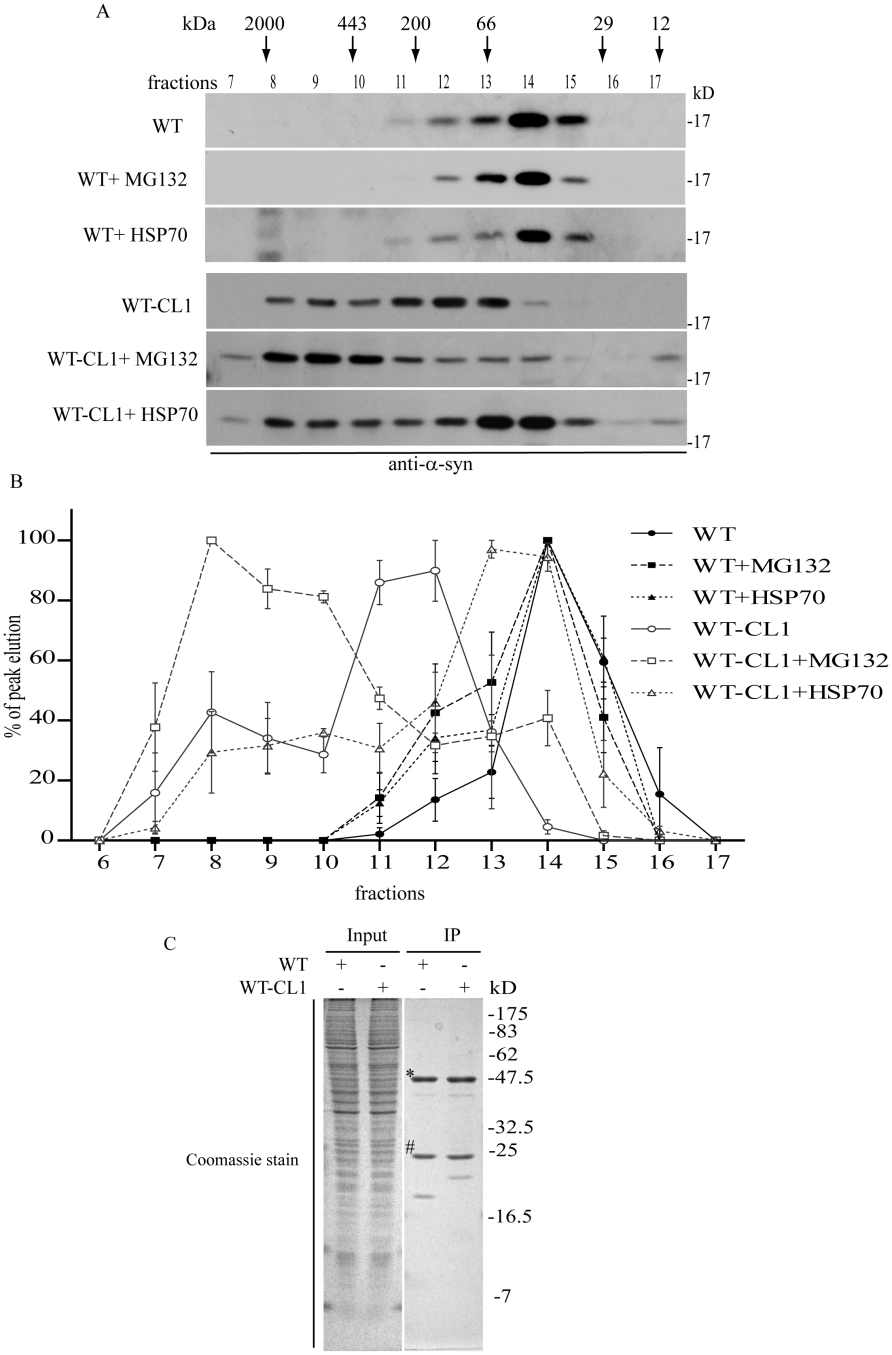
Formation of high molecular weight (HMW) α-syn oligomers is linked to increased cytotoxicity. (A) SHSY5Y cells expressing WT or WT-CL1 α-syn under different conditions as indicated, were harvested and subjected to size exclusion chromatography (SEC) analysis. Fractions eluted were subjected to Western blot analysis to determine the amount and elution profile of α-syn (B). The elution profile as in (A) for 3 independent experiments were quantified by optical densitometry and presented as the percentage of the peak elution. (C) SHSY5Y cells expressing WT or WT-CL1 α-syn were subjected to immunoprecipitation and the immunoprecipitates were subjected to SDS-PAGE followed by Coomassie staining (* and # were the heavy and light chains of antibody).

Although the filter retardation assay can measure the levels of α-syn aggregation in cells, it does not provide information regarding the changes in the oligomerization of α-syn. Therefore, we used the size exclusion chromatography (SEC) to monitor the oligomerization of WT and WT-CL1 α-syn in SHSY5Y cells. WT α-syn expression in SHSY5Y cells was mainly eluted at a major molecular size of approximately 50–60 kDa, which is consistent with data from several previous studies (Figure 5 A) [38]–[41]. WT-CL1 α-syn was eluted at fractions between the molecular sizes of 200 and 66 kDa (Figure 5A). Interestingly, the attachment of CL1 to α-syn enhanced its oligomerization as significant higher molecular weight (HMW) oligomers of WT-CL1 α-syn were detected (Figure 5 A & B) (F = 17.47; p<0.0001, Two way ANOVA). To confirm that the elution of WT-CL1 α-syn at the higher molecular weight was not caused by binding to other cellular proteins, we immunoprecipitated the WT and WT-CL1 α-syn in SHSY5Y cells and did not observed any major co-immunoprecipitated proteins (Figure 5C). This supported the notion that WT-CL1 α-syn had a higher tendency to form HMW oligomers in SHSY5Y cells. Our cellular data showed that treatment of MG132 could aggravate the toxicity induced by WT and WT-CL1 α-syn, which was associated with an increased in α-syn aggregation (Figure 4). In contrast, coexpression of HSP70 reduced WT-CL1 α-syn-induced toxicity associated with reduction in α-syn aggregation (Figure 4). To determine if oligomerization of WT and WT-CL1 α-syn were also affected in the treatment of MG132 and coexpression of HSP70, we used SEC to monitor changes in oligomerization of WT and WT-CL1 α-syn in SHSY5Y cells. MG132 treatment significantly enhanced WT α-syn oligomerization (Figure 5 A & B) (F = 2.15; p<0.05, Two way ANOVA), but coexpression of HSP70 did not have any effects on its oligomers formation (Figure 5 A & B) (F = 1.19; p = n.s., Two way ANOVA). For SHSY5Y cells expressing WT-CL1 α-syn, MG132 treatment significantly enhanced the oligomerization of WT-CL1 α-syn (Figure 5 A & B) (F = 8.70; p<0.0001, Two way ANOVA). In contrast, coexpression of HSP70 significantly reduced the WT-CL1 α-syn oligomerization (Figure 5 A & B) (F = 9.69; p<0.0001, Two way ANOVA). Overall, these data suggested that conditions that aggravated α-syn toxicity enhanced its oligomerization, whereas factor that protected against α-syn cytotoxicity reduced the oligomerization of α-syn.

### CL1 Enhances the Degeneration of Dopaminergic Neurons by α-syn in an Age-dependent Manner

**Figure 6:**
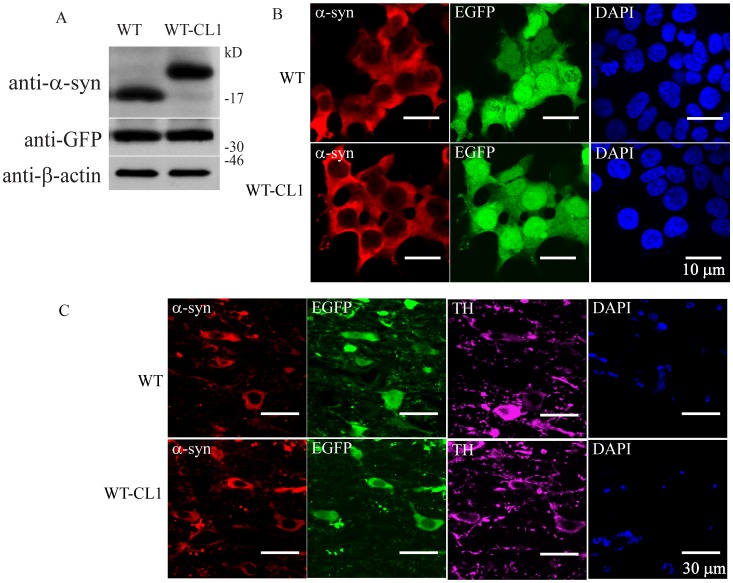
Establishment of an adenovirus expression system that expresses WT or WT-CL1 α-syn. (A) SHSY5Y cells infected with adenovirus expressing WT or WT-CL1 α-syn were subjected to Western blot analysis. Similar expression level of WT or WT-CL1 α-syn was observed. (B) SHSY5Y cells infected with adenovirus expressing WT or WT-CL1 α-syn were subjected to Immunocytochemistry. (C) Dopaminergic neurons of mice in the SNc were infected with adenovirus expressing WT or WT-CL1 α-syn and subjected to immunohistochemistry. Sections at the SNc were stained with antibodies against TH and human α-syn.

**Figure 7:**
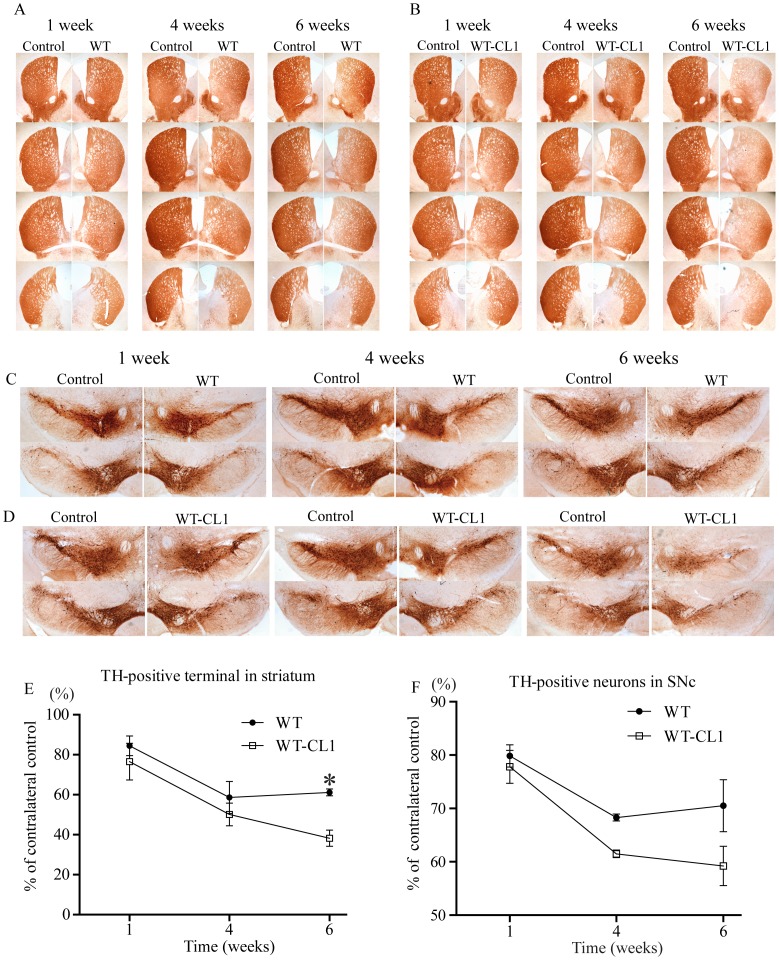
CL1 enhances the degeneration of dopaminergic neurons by α-syn in an age-dependent manner. Mice were injected with control or WT α-syn (at the contralateral side) expressing adenovirus in the MFB and were sacrificed 1, 4 and 6 weeks after the injection. The brains were harvested and the striatum (A) and SNc (C) were subjected to immunohistochemistry with anti-TH antibodies. Mice were injected with control or WT-CL1 α-syn (at the contralateral side) expressing adenovirus at the MFB and were sacrificed after 1, 4 and 6 weeks of injection. The brains were harvested and the striatum (B) and SNc (D) were subjected to immunohistochemistry with anti-TH antibodies. (E) The striatal TH terminals injected with WT or WT-CL1 α-syn expressing adenovirus as in (A) and (B) were quantified by optical densitometry. The density of the striatal TH terminals was presented as percentage intensity of the contralateral side injected with the control adenovirus. Four animals for each time point were measured. (F) The TH positive neurons in SNc injected with WT or WT-CL1 α-syn expressing adenovirus as in (C) and (D) were quantified in a blind manner. The TH positive neurons were presented as percentage of the contralateral side injected with the control adenovirus. Four animals for each time point were measured.

**Figure 8:**
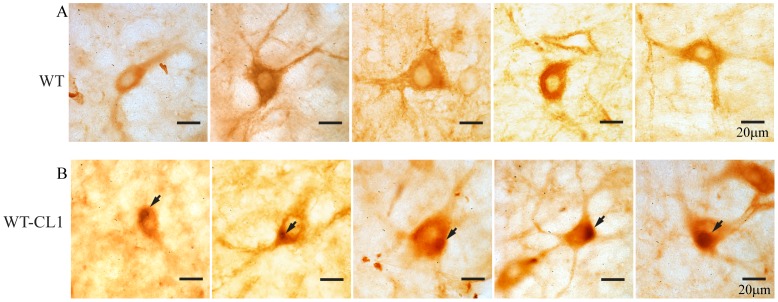
Formation of Lewy body-like aggregates in neurons in the SNc expressing WT-CL1 α-syn. Mice injected with WT (A) or WT-CL1 (B) α-syn expressing adenovirus were sacrificed after 3 months and neurons at the SNc were subjected to immunohistochemistry with the human specific anti-α-syn antibody LB509. Lewy body-like perinuclear aggregates were observed in the neurons of SNc.

Our *in vitro* and cellular results showed that CL1 could enhance the oligomerization and aggregation of α-syn. To determine if WT-CL1 α-syn could aggravate α-syn-induced neurodegeneration *in vivo*, we generated adenovirus that expressed WT or WT-CL1 α-syn and infected dopaminergic neurons in mice. First, we generated the WT and WT-CL1 α-syn expressing adenovirus and tested their expression efficiency in SHSY5Y cells, and found that both WT and WT-CL1 α-syn were expressed at a similar level (Figure 6 A & B). We also tested the infection of tyrosine hydroxylase (TH) neurons in the substantia nigra pars compacta (SNc) by stereotactically injecting WT and WT-CL1 α-syn to the medial forebrain bundle (MFB) and found that these viruses could effectively infect TH neurons (Figure 6C). To determine if CL1 could aggravate the toxicity towards TH neurons induced by α-syn, we injected WT and WT-CL1 α-syn expressing adenovirus stereotactically into mice at the MFB. Then, we monitored the degeneration of TH neurons by measuring the TH immunoreactivity in the striatum and quantifying TH positive neurons in the SNc 1, 4 and 6 weeks after injection. Infection of adenovirus expressing both WT and WT-CL1 α-syn led to the degeneration of the TH neuronal terminal in the striatum (Figure 7 A, B & E) (F = 14.82; p<0.0001, Two way ANOVA). WT-CL1 α-syn significantly enhanced the degeneration of TH terminal compared to WT α-syn in an age-dependent manner (Figure 7 A, B & E) (F = 6.92; p<0.05, Two way ANOVA). Similarly, expression of both WT and WT-CL1 α-syn led to degeneration of the TH positive neurons in the SNc (Figure 7 C, D & F) (F = 14.87; p<0.0001, Two way ANOVA). In addition, WT-CL1 α-syn significantly enhanced the degeneration of TH neurons compared to WT α-syn in an age-dependent manner (Figure 7 C, D & F) (F = 7.74; p<0.05, Two way ANOVA). Because our *in vivo* data showed that WT-CL1 α-syn increased the toxicity of α-syn, we used the human specific anti-α-syn antibody to determine if α-syn aggregation was prominent in the SNc. In mice injected with WT α-syn-expressing adenovirus, we observed a general cytoplasmic staining in neurons at the SNc (Figure 8 A). In contrast, in mice injected with WT-CL1 α-syn-expressing adenovirus, in addition to cytoplasmic staining, perinuclear aggregates that resembled LBs were observed (Figure 8 B). In summary, these results showed that WT-CL1 α-syn could enhance the degeneration of dopaminergic neurons and formation of Lewy body-like aggregates in SNc.

## Discussion

Previous studies have suggested that oxidative stress or post-translational modification such as nitration and phosphorylation enhance the oligomerization and aggregation of α-syn which can subsequently cause the degeneration of dopaminergic neurons [22]–[24], [42]–[46]. However, the primary toxic species of α-syn has not been well defined. Initial studies suggested that prominent α-syn aggregates such as Lewy bodies and neurites are the components that cause the neuronal degeneration, but studies have also suggested that protein aggregates might be protective [22]–[25]. The understanding of the role of α-syn aggregation in PD is crucial for the development of novel treatments that target α-syn aggregation. In this study, we addressed the role of α-syn aggregation and cytotoxicity by using the characteristic features of the CL1 peptide. Attachment of CL1 sequestered both WT and mutant α-syn to the detergent-insoluble fraction of the cells and facilitated their aggregation. With this approach, we were able to demonstrate a direct correlation between α-syn aggregation and reduced cell viability. Interestingly, while WT-CL1 α-syn had a shorter half-life as determined by the pulse-chase experiment, the steady state protein level remained comparable to WT α-syn (Figure 1 A & B). This might suggest that the WT and WT-CL1 expression levels were different and might contribute to the difference in α-syn aggregation and cell viability. However, we also found that coexpression of HSP70 or treatment of MG132, which affected α-syn aggregation and cell viability, did not affect the overall expression levels of WT and WT-CL1 α-syn (Figure S1 A & B), suggesting that the expression levels were not a contributing factor in our study. In addition, the CL1-enhanced aggregation and increased toxicity of α-syn was specific as attachment of CL1 to β-syn and Htt-Q23 enhanced their aggregation but did not affect their cytotoxicity. From the SEC, we found that attachment of CL1 enhanced α-syn oligomerization and this was further increased in cells treated with MG132. In contrast, coexpression of HSP70 significantly reduced the oligomerization of α-syn. Interestingly, we observed that coexpression of HSP70 could reduce the size of WT-CL1 as determined by SEC, suggesting that HSP70 could possibly modulate the overall conformation of WT-CL1.

Overall, these results suggest that enhanced oligomerization and aggregation of α-syn is associated with an increased in α-syn induced-cytotoxicity. From our *in vivo* animal model of PD, we found that attachment of CL1 facilitated the formation of Lewy body-like α-syn aggregates, but at the same time it enhanced the toxicity induced by α-syn in an age-dependent manner. These results suggest that enhanced aggregate formation might not be protective, contrary to some of the suggestions in previous studies [22]–[24], [26]–[28]. In this study, we have also established a new animal PD model by expressing WT-CL1 α-syn in the dopaminergic system and caused an age-dependent degeneration of TH neurons in association with the formation of intraneuronal Lewy body-like aggregates. This model will be a valuable tool for studying how α-syn oligomerization and aggregation contribute to neurodegeneration in PD.

## Materials and Methods

### Chemicals, Antibodies, Plasmids and Cell Lines

All chemicals were purchased from Sigma (St. Louis, MO) unless otherwise stated. Antibodies used for immunoblotting included the following: mouse anti-myc (Roche), mouse anti-HA (Roche), mouse anti-human α-syn phospho (Ser129) (anti-pS129α-syn) (Wako Chemicals), mouse anti-α-syn (BD Transduction Laboratories), mouse anti-human α-syn (LB509; Invitrogen), mouse anti-β-actin, rabbit anti-tyrosine hydroxylase (anti-TH; Abcam), rabbit anti-GFP (Abcam), rabbit anti-HA (Sigma) and rabbit anti-α-syn. pRK5 HA-ubiquitin (HA-Ub), pRK5 WT, E46K and A53T α-syn were generous gifts from Professor Ted Dawson (Johns Hopkins University, Baltimore, MD, USA). CL1 (ACKNWFSSLSHFVIHL) [30], [31] was fused to the C-terminal of WT, E46K and A53T α-syn by fusion PCR and cloned into pRK5 vector between the SalI and NotI sites to generate WT-CL1, E46K-CL1 and A53T-CL1 α-syn. HSPA1A (HSP70) β-synuclein (β-syn) were PCR from the SuperScript human brain cDNA library (Invitrogen) and cloned into pRK5-myc by SalI and NotI. A huntingtin fragment (1–171) with 23 CAG repeats (Htt-Q23) was a generous gift from Prof. Edwin Chan (CUHK, HKSAR, China) and was subcloned into pRK5-myc between the SalI and NotI sites. β-syn-CL1 and Htt-Q23-CL1 were generated by fusion PCR and cloned into pRK5-myc vector between SalI and NotI. For recombinant protein production WT and WT-CL1 α-syn were subcloned into pGEX-4T-2 and pET-28(c) between SalI and NotI. For the generation of adenovirus, WT and WT-CL1 α-syn with IRES-EGFP (WT-IRES-EGFP and WT-CL1-IRES-EGFP) were cloned into the modified pENTR™/U6 (Invitrogen) in which the U6 promoter was replaced by a CMV promoter to generate pENTR™/CMV WT-IRES-EGFP and pENTR™/CMV WT-CL1-IRES-EGFP. The adenovirus expression clones were then generated by recombination according to the manufacturer’s instructions. The SHSY5Y cell line was acquired from ATCC, and the HEK293A cell line was acquired from Invitrogen.

### Recombinant Protein Purification and Antibody Generation

pGEX-4T-2, pGEX-4T-2-WT α-syn, pGEX-4T-2-WT-CL1 α-syn, pET-28(c)-WT α-syn and pET-28(c)-WT-CL1 α-syn were transformed into Rosetta (DE3) pLys *E. coli* (Novagen) to generate recombinant protein GST, GST-WT α-syn, GST-WT-CL1 α-syn, His-WT α-syn and His-WT-CL1 α-syn respectively. Induction was performed with 0.2 mM IPTG at 16°C for 16–18 h. GST-tagged recombinant proteins were purified using GSH-Sepharose (GE Healthcare) and His-tagged proteins were purified using Ni-NTA Sepharose (GE Healthcare) according to the manufacturer’s instruction. Purified His-WT α-syn in PBS was mixed with equal volume of Freund’s adjuvant to form an emulsion. The mixture was used to immunize a New Zealand white rabbit. Anti-α-syn antibody was purified from serum with GST-WT α-syn conjugated to CNBr-activated Sepharose (GE Healthcare) according to manufacturer’s instruction.

### Cell Culture and Transfection

SHSY5Y and HEK293A cells were maintained in Dulbecco’s Modified Eagle Medium (Invitrogen) supplemented with 10% FBS (Invitrogen) and antibiotics in a 37°C incubator with 5% CO_2_. Transient transfection of DNA to SHSY5Y or HEK 293A cells was performed using Lipofectamine (Invitrogen) and PLUS reagents (Invitrogen) according to the manufacturer’s instructions. Primary cortical neurons from ICR mice were prepared from E16 embryos. Cortical neurons were dissected out and digested with TrypLE Express (Invitrogen) in Hank’s Balanced Salt Solution (HBSS) for 15 min. Neurons were then washed twice with HBSS and resuspended in Neurobasal Medium (NB) supplemented with 2% B-27 and 0.5 mM glutamax with antibiotics (Invitrogen), and subsequently seeded on poly-L-lysine coated coverslips. Neurons were transfected with Lipofectamine 2000 (Invitrogen) according to the manufacturer’s instructions.

### Preparation and Analysis of Proteins in the Soluble and Insoluble Fractions in SHSY5Y Cells

SHSY5Y cells were transfected with plasmids as indicated and harvested 48 h after transfection by two washes with ice cold PBS. They were subsequently lysed with Triton soluble buffer (1% Triton X-100, 0.5 µM EDTA, 1 mM leupeptin, 1 mM aprotinin, 1 mM benzamidine and 10 mM PMSF in TBS) and rotated at 4°C for 1 h. After rotation, cell lysates were centrifuged at 14,000 rpm for 10 min and the supernatant was collected as Triton soluble fractions. Insoluble fractions were prepared by resuspending the pellets of the lysate in SDS soluble buffer (1% Triton X-100, 2% SDS, 1% Na deoxycholate, 1% NP40, 0.5 µM EDTA, 1 mM leupeptin, 1 mM aprotinin, 1 mM benzamidine and 10 mM PMSF in TBS) and subjecting them to sonication. The protein concentration of each sample was determined using a BCA protein assay kit (Pierce), and samples were then subjected to Western blot analysis.

### Cycloheximide Pulse Chase Analysis

SHSY5Y cells were transfected with plasmids as indicated and 48 h after transfection, cells were treated with 10 µM cycloheximide and harvested at different time point as indicated. Cells were then lysed with lysis buffer (1% Triton X-100, 2% SDS, 1% Na deoxycholate, 1% NP40, 0.5 µM EDTA, 1 mM leupeptin, 1 mM aprotinin, 1 mM benzamidine and 10 mM PMSF in TBS) and subjected to sonication. The protein concentration of each sample was determined using the BCA protein assay kit (Pierce), and subsequently the samples were subjected to Western blot analysis. The optical density of bands was measured by the gel analysis function of ImageJ software (National Institute of Mental Health) normalized to β-actin.

### Lipid Sedimentation Assay

The lipid sedimentation assay was performed as described [47] with small modification. Liposome was prepared by suspending 2 mg/ml of bovine brain lipid extracts (Folch fraction I) in buffer with 20 mM HEPES (pH 7.4), 150 mM NaCl, and 1 mM DTT. GST, GST-WT α-syn or GST-WT-CL1 α-syn recombinant protein (5 µM) was then incubated with 0.5 mg liposome in 100 µl buffer at 4°C for 2 h and centrifuged at 140,000 g for 15 min. The supernatant and the pellet were collected and subjected to SDS-PAGE and stained with Coomassie blue.

### Filter Retardation Assay and Cell Death Analysis

SHSY5Y cells were transfected with different plasmids as indicated and treated with vehicle or MG132 (5 µM) 24 h after transfection. After 24 h treatment, cells were harvested and half of the cells were subjected to filter trap retardation assay and the other half was subjected to the cell viability assay. For the filter retardation assay, SHSY5Y cells were washed once with PBS, lysed with lysis buffer (TBS, 2% SDS, 1% NP40, 150 mM NaCl, 10% glycerol, 20 mM NaF, 0.5 µM EDTA, 1 mM leupeptin, 1 mM aprotinin, 1 mM benzamidine and 10 mM PMSF) and subjected to sonication. The protein concentration of samples was determined using the BCA Protein Assay Kit (Pierce) and 50 µg of protein from each sample was loaded to a 0.2 µm nitrocellulose membrane (Bio-Rad) in a Bio-Dot Micro-filtration Apparatus (Bio-Rad), followed by Western blot analysis. For cell viability analysis, SHSY5Y cells were washed once with PBS, and then resuspended in FBS containing DMEM. Cell viability was determined by Trypan blue exclusion assay. The number of live cells and Trypan blue stained cells were scored and quantified in a double-blinded manner.

### Electron Microscopy (EM)

Purified recombinant His-WT α-syn and His-WT-CL1 α-syn (3 µM) were incubated at 37°C for 2 weeks with shaking (250 rpm). After incubation, 10 µl of protein was spotted on the Formvar-coated, carbon-stabilized copper grid (400 mesh) (SPI Supplies). The samples were allowed to adsorb for 30 min at room temperature, washed with 100 µl Millipore-filtered water and then negatively stained with 1% aqueous uranyl acetate. Afterwards, the samples were washed with 100 µl Millipore-filtered water and dried with compressed air. EM images were obtained with a JEOL 2010 TEM (JEOL Ltd.) at an accelerating voltage of 120 kV.

### Atomic Force Microscope (AFM) Imaging

Purified His-WT α-syn and His-WT-CL1 α-syn at a concentration of 3 µM were incubated for 1 month at 4°C or 37°C with or without 10 mM Congo red by vigorous shaking (250 rpm). Before AFM imaging, the protein samples were gently mixed to resuspend any aggregates and then 5 µl of protein was spotted on freshly cleaved mica (Ted Pella). The samples were allowed to adsorb to the mica for 10 min at room temperature and then washed with 1 ml Millipore-filtered water and dried with compressed air. Images were obtained with a Nanoscope Multimode 8 AFM using the ScanAsyst in air tapping mode (Veeco/Digital Instruments) with an etched silicon NanoProbes (model FESP, Digital Instruments). The raw image data were analyzed using NanoScope Analysis software (Veeco/Digital Instruments).

### Immunocytochemistry *of α-syn Aggregation in SHSY5Y Cells and Neurons*

For SHSY5Y cells, 48 h after transfection of plasmids as indicated, cells were washed once with PBS, then fixed with 4% ice cold paraformaldehyde (PFA) for 20 min and permeabilized with 0.2% Triton X-100 for 10 min. Cells were then blocked with 10% normal donkey serum (NDS) for 1 h and subsequently incubated with mouse anti-α-syn antibody overnight at 4°C. After overnight incubation, cells were incubated with anti-mouse secondary antibodies, followed by 4′-6-Diamidino-2-phenylindole (DAPI) for nucleus visualization. For double staining experiment, SHSY5Y cells transfected with WT-CL1 α-syn and HA-Ub were sequentially incubated with mouse anti-α-syn and rabbit anti-HA antibodies. For the staining of phospho-Ser 129 α-syn, SHSY5Y cells transfected with WT-CL1 α-syn were sequentially incubated with rabbit anti-α-syn and mouse anti-pS129 α-syn antibodies. For primary cortical neurons, neurons were transfected with plasmid as indicated at 3 days *in vitro* (3DIV). Six days after transfection, neurons were harvested and stained for human α-syn as described. All pictures were taken with a confocal microscope (LSM510, Zeiss).

### Size Exclusion Chromatography

SHSY5Y cells transfected with plasmid and treated as indicated were harvested 48 h after transfection. Cells were then washed once with PBS and lysed with 0.5% Triton X-100 in TBS at 4°C for 1 h. After incubation, lysates were centrifuged at 14,000 rpm for 10 min and filtered with a 0.45 µm filter (*Millipore*). After filtration, 3 mg of cell lysate was injected into a superdex 200 (10/23HR) column (GE healthcare) and the fractions were collected and subjected to Western blot analysis. The optical density of each fraction was measured by Image J software (National Institute of Mental Health) and the elution profiles were presented as the percentage of the peak elution.

### Immunoprecipitation

SHSY5Y cells transfected with myc-tagged WT or WT-CL1 α-syn were harvested 48 h after transfection by washing with ice cold PBS and then lysed with immunoprecipitation (IP) buffer (1% Triton X-100, 1 mM aprotinin, 1 mM leupeptin, 1 mM benzamidine, 10 mM PMSF in PBS). The lysates were then rotated at 4°C for 1 h and subsequently centrifuged at 14,000 rpm for 10 min. The supernatant was incubated with 2 µg anti-myc antibody and protein A agarose (GE Healthcare) at 4°C for 2 h. After incubation, the agarose was pelleted at 2,000 rpm and washed 3 times with IP buffer. The precipitates were then subjected to SDS-PAGE and stained with Coomassie blue.

### Adenovirus Production and Viral Analysis

Adenoviral particles were produced by HEK293A cells following the manufacturer’s instruction (Invitrogen). In brief, adenoviral vectors, pENTR™/CMV WT-IRES-EGFP and pENTR™/CMV WT-CL1-IRES-EGFP, were transfected into HEK293A cells and grown until cells were detached from the culture plate. The culture medium and the cells were then collected and subjected to 3 freeze/thaw cycles between −80°C and 37°C. Samples were centrifuged at 4,000 g for 15 min and the supernatant containing the adenovirus virus was transferred to a new tube and stored at −80°C. The infectious unit (IFU) of the virus was determined by infecting HEK293 cells followed by FACS analysis as described [48].

### Stereotaxic Injection of Adenovirus in Mice

All animals were housed, cared for, and experiments conducted according to relevant national and international guidelines. The animal protocols used have been reviewed and approved by the Animal Ethics Committee of The Hong Kong University of Science and Technology. Adenovirus with a titer of 2×10^9^ IFU/ml (2 µl) was stereotaxically injected into the medial forebrain bundle (MFB) of C57/BL6 male mice (3 month old) with the following coordinates; −0.12 mm anterior-posterior, +/−0.12 mm mediolateral, and −0.45 mm dorsoventral. Adenovirus expressing WT-α-syn-IRES-EGFP or WT-α-syn-CL1-IRES-EGFP was injected on the right side with adenovirus expressing EGFP as the control injected on the contralateral side.

### Immunohistochemistry

Mice were deeply anesthetized with pentobarbital and were perfused with PBS and 4% PFA in PBS. The mouse brains were then harvested and post-fixed overnight in 4% PFA and subsequently cryoprotected by incubation in 30% sucrose. Coronal sections 40 µm in thickness were prepared using a cryostat (Leica). For fluorescence detection of WT or WT-CL1 α-syn expression in tyrosine hydroxylase (TH) neurons, sections were incubated with mouse anti-human α-syn and rabbit anti-TH antibody using the same procedures as in the immunocytochemistry study. Pictures were taken with a confocal microscope (LSM510, Zeiss) using a 40 × Plan Apo Oil NA 1.4 DIC objective (Zeiss). For the immunostaining using the ABC kit (Vector Laboratories), sections were first quenched for 10 min in 3% H_2_O_2_ and 10% methanol, then washed with TBS and blocked with 3% normal goat serum. Sections were then incubated with rabbit anti-TH or mouse anti-human α-syn antibodies overnight at 4°C. After overnight incubation, sections were washed and incubated with biotinylated secondary antibodies (goat anti-rabbit or goat anti-mouse; Vector Laboratories) for 2 h. After washing, the sections were stained with the ABC kit according to manufacturer’s instructions. Stained sections were mounted and dried on gelatinized slides, dehydrated and coverslipped.

### Quantification of TH Terminal Density and Neurons in the Striatum and SNc

For quantification of TH terminal density and neurons in the striatum and SNc, mice from 1 week, 4 weeks and 6 weeks time point (4 mice per time point) were sacrificed and 5 striatal and SNc sections from each mouse were stained with rabbit anti-TH antibody using the ABC kit (Vector Laboratories) as described. Pictures for striatum and SNc were taken using a 2 × plan apo objective (Leica). The optical density of the TH terminal was measured using the ImageJ software (National Institute of Mental Health). The optical density of the TH terminals was calculated as the percentage of their contralateral side. The TH positive neurons in the SNc were quantified in a blind manner and were calculated as the percentage of their contralateral side.

### Data and Statistical Analysis

Data were expressed as SEM, and the student t-test, one way and two way ANOVA were used to determine statistical significance.

## Supporting Information

Figure S1 CL1 enhanced aggregation and increased toxicity in α-syn is specific. The total protein expression levels of WT (A) and WT-CL1 (B) α-syn were not affected by coexpression of HSP70 or treatment with MG132 CL1 also enhanced aggregation in β-syn and Htt-Q23 (C), but did not affect their toxicity (D).
(TIF)
