# Supplementary figures and images for: The Role of Alpha-Synuclein Oligomerization and Aggregation in Cellular and Animal Models of Parkinson’s Disease
Source: PLoS One. 2012 Jun 12;7(6):e38545. doi: 10.1371/journal.pone.0038545 (PMC3373518; doi:10.1371/journal.pone.0038545)

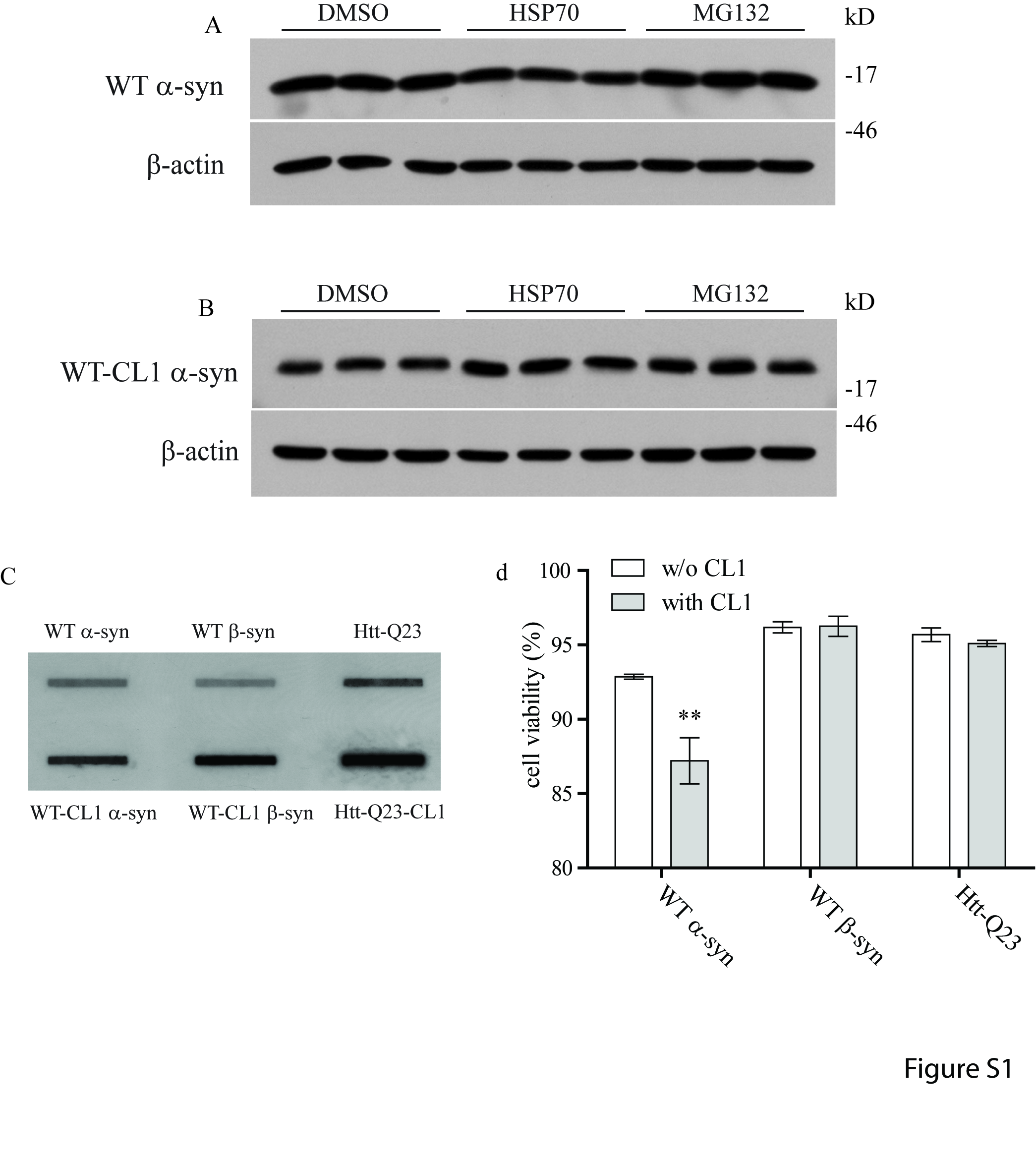

Supplement: Figure S1 — CL1 enhanced aggregation and increased toxicity in α-syn is specific. The total protein expression levels of WT (A) and WT-CL1 (B) α-syn were not affected by coexpression of HSP70 or treatment with MG132 CL1 also enhanced aggregation in β-syn and Htt-Q23 (C), but did not affect their toxicity (D). (TIF) [file pone.0038545.s001.tif]
